# Fracture resistance of lithium disilicate occlusal veneers fabricated with CAD/CAM and luted to dentin: a systematic review

**DOI:** 10.21142/2523-2754-1402-2026-291

**Published:** 2026-04-04

**Authors:** Jennifer Stephanie Llompart-Delgado, Pedro Luis Tinedo-Lopez, Martín Andrés Chávez-Méndez

**Affiliations:** 1 Carrera de Estomatología, Universidad Científica del Sur. Lima, Perú. jllompart@cientifica.edu.pe ptinedo@cientifica.edu.pe Carrera de Estomatología Universidad Científica del Sur Lima Perú jllompart@cientifica.edu.pe ptinedo@cientifica.edu.pe; 2 Departamento de Implantología Oral, Departamento de Posgrado de Estomatología, Universidad Científica del Sur. Lima, Perú. Universidad Científica del Sur Departamento de Implantología Oral Departamento de Posgrado de Estomatología Universidad Científica del Sur Lima Peru; 3 Grupo de investigación Oral Peri-implant, Dirección de Investigación, Desarrollo e Innovación (DGIDI), Carrera de Estomatología, Universidad Científica del Sur. Lima, Perú. mchavezme@cientifica.edu.pe Universidad Científica del Sur Grupo de investigación Oral Peri-implant Dirección de Investigación, Desarrollo e Innovación (DGIDI) Universidad Científica del Sur Lima Peru mchavezme@cientifica.edu.pe

**Keywords:** lithium disilicate, occlusal veneers, CAD/CAM, dentin bonding, fracture resistance, in vitro studies, disilicato de litio, carillas oclusales, CAD/CAM, adhesión dentina, resistencia a la fractura, estudios in vitro

## Abstract

**Background:**

Lithium disilicate occlusal veneers offer a conservative solution for restoring posterior tooth wear, especially when bonded to dentin. CAD/CAM technology has enabled precise fabrication of these restorations, but concerns remain regarding their fracture resistance in demanding clinical scenarios. Our objective is to evaluate the fracture resistance of CAD/CAM-fabricated lithium disilicate occlusal veneers luted to dentin through a systematic review of in vitro studies.

**Methods:**

A systematic search was conducted in PubMed, Web of Science, Scopus, and Embase for in vitro studies published in English, Spanish, or Portuguese over the last 10 years up to 2025. Inclusion criteria focused on experimental studies assessing fracture resistance of lithium disilicate occlusal veneers bonded to dentin. Risk of bias was assessed using the QUIN tool.

**Results:**

Eight studies met the inclusion criteria. All used human teeth and evaluated veneers with thicknesses ranging from 0.5 mm to 2.5 mm. CAD/CAM lithium disilicate veneers consistently demonstrated high fracture resistance values, exceeding typical posterior bite forces. Fracture resistance was positively influenced by increased thickness and the use of immediate dentin sealing. The aging protocols (thermocycling or water storage) showed limited impact on outcomes. All studies were rated as low risk of bias.

**Conclusion:**

Lithium disilicate occlusal veneers fabricated with CAD/CAM and luted to dentin show excellent fracture resistance. A thickness between 0.8 mm and 1.5 mm is recommended for optimal strength, especially when immediate dentin sealing is applied. Further research should explore adhesive protocols to enhance dentin bonding.

## RESUMEN

**Objetivo:**

Las carillas oclusales de disilicato de litio ofrecen una solución conservadora para la restauración de desgaste dental posterior, especialmente cuando son cementadas en dentina. La tecnología CAD/CAM ha hecho posible la fabricación de estas restauraciones; sin embargo, existe aún preocupación en lo referido a la resistencia a la fractura en escenarios clínicos demandantes. Nuestro objetivo es evaluar la resistencia a la fractura de carillas oclusales de disilicato de litio fabricadas en CAD/CAM cementadas en dentina mediante una revisión sistemática de estudios *in vitro*.

**Método:**

Una revisión sistemática fue realizada en PubMed, Web of Science, Scopus y Embase, en busca de estudios in vitro publicados en los últimos 10 años en inglés, español o portugués. Los criterios de inclusión se centraron en estudios experimentales que evaluaron la resistencia a la fractura de carillas oclusales de disilicato de litio adheridas a la dentina. El riesgo de sesgo se evaluó mediante la herramienta QUIN.

**Resultados:**

Ocho estudios cumplieron los criterios de inclusión. Todos utilizaron dientes humanos y evaluaron carillas con espesores que oscilaban entre 0,5 mm y 2,5 mm. Las carillas de disilicato de litio CAD/CAM demostraron consistentemente altos valores de resistencia a la fractura, al superar las fuerzas de mordida posteriores típicas. La resistencia a la fractura se vio influenciada positivamente por el aumento del espesor y el uso de sellado de dentina inmediato. Los protocolos de envejecimiento (termociclado o almacenamiento en agua) mostraron un impacto limitado en los resultados. Todos los estudios se clasificaron como de bajo riesgo de sesgo.

**Conclusión:**

Las carillas oclusales de disilicato de litio fabricadas mediante CAD/CAM cementadas a la dentina muestran una excelente resistencia a la fractura. Se recomienda un grosor de entre 0,8 mm y 1,5 mm para una resistencia óptima, especialmente al aplicar sellado dentinario inmediato. Se recomiendan futuras investigaciones para explorar protocolos adhesivos que mejoren la adhesión a la dentina.

## INTRODUCTION

Due to technological advancements, restorative dentistry has developed new materials that allow the rehabilitation of complex clinical cases involving severe tooth wear, without sacrificing a significant amount of dental tissue ^1^^-^^3^. Although minimal preparation was traditionally associated with facial veneers, this concept has now been extended to occlusal veneers, which require only minimal tooth reduction and provide little to no mechanical retention. In such cases, it is essential to round any sharp angles on the surface designated for veneer placement and preserve the remaining enamel to ensure optimal bonding ^2^.

The wide variety of materials and minimal thicknesses available for these restorations enables their application at different stages of a treatment plan ^4^. The integration of novel materials with CAD/CAM technology, together with continuous advancements in adhesive protocols-such as immediate dentin sealing-has made it possible to restore severely worn teeth without further compromising the remaining tooth structure or pulpal vitality ^4^.

Only ceramics and composite resins adhere to the biomimetic principles of tissue preservation and esthetics. The use of ceramics as enamel substitutes is highly recommended ^5^^,^^6^, with the choice of material primarily depending on its strength, thickness ^7^, and ability to bond effectively to adjacent dental substrates ^8^^,^^9^, thereby mimicking the enamel-dentin junction. The development of stronger ceramics that remain both etchable and machinable has broadened the indications for adhesive ceramic restorations ^10^^,^^11^.

Among these materials, lithium disilicate-a glass-ceramic-stands out for its high flexural strength, biocompatibility, translucency, and excellent esthetic properties ^12^. Studies on the fracture resistance of occlusal veneers fabricated from different materials aim to identify the most suitable options for efficiently restoring cases of dental wear ^13^^,^^14^. However, these investigations vary considerably in methodology, ranging from in vitro experiments to three-dimensional simulations ^13^^-^^16^.

Amesti-Garaizabal et al. ^17^ conducted a systematic review on the fracture resistance of partial indirect restorations fabricated with CAD/CAM technology, analyzing restorations from inlays to overlays using diverse materials such as machined resins and ceramics. Nevertheless, their review did not include occlusal veneers, nor did it distinguish the type of dental substrate involved in the restorations.

The substrate to which a restoration is bonded depends on the clinical condition of the tooth, with exposed dentin representing one of the greatest challenges ^15^. Some studies ^18^^,^^19^ have evaluated the fracture resistance of occlusal veneers of varying thicknesses on favorable substrates such as enamel. However, as this is a relatively new restorative alternative, no consensus has yet been reached regarding their use, even though they are increasingly incorporated into comprehensive rehabilitative treatments.

Systematic reviews play a crucial role in synthesizing existing evidence and providing critical analyses of specific topics. To date, only one systematic review ^20^ has assessed bonded partial restorations on enamel substrates. Nevertheless, further research is required to focus specifically on occlusal veneers.

Therefore, the aim of the present study was to analyze the outcomes of in vitro studies on CAD/CAM-manufactured lithium disilicate occlusal veneers and to determine their fracture resistance when cemented to dentin.

## MATERIALS AND METHODS

In accordance with methodological recommendations for systematic reviews in the health sciences-which advise limiting the search period when technological advancements are expected to directly influence outcomes (PRISMA, Cochrane Handbook) ^21^-articles published within the last 10 years up to 2025 were analyzed. The search focused on experimental in vitro studies published in English, Spanish, or Portuguese. The included studies specifically evaluated the fracture resistance of lithium disilicate occlusal veneers fabricated using CAD/CAM technology and luted to dentin.

The systematic review was conducted using the following electronic databases: MEDLINE via PubMed, Web of Science (WOS), Scopus, and Embase. The review was registered at Universidad Científica del Sur under the registration number POS-53-2023-00277, and the protocol was also registered in PROSPERO (International Prospective Register of Systematic Reviews) under the code CRD42024533901.

### Search strategy

The systematic review was performed using several databases (Table 1). Different keywords, Boolean operators (OR, AND), and MeSH terms were used for each database as follows: "occlusal veneers" OR "occlusal veneer" OR "onlay" OR "onlays" OR "occlusal veneer*" AND "CAD/CAM" AND "dentin" AND "lithium disilicate" OR "ceramics" OR "ceramic" AND "fracture" OR "strength fracture"

**Table 1:** Search strategy on data bases

Data base (strategy)		
1.	PubMed:	16
(((((((("occlusal veneers") OR ("occlusal veneer")) OR ("onlay")) OR ("onlays")) OR ("occlusal veneer*")) AND ("CAD/CAM")) AND ("dentin")) AND (((("lithium disilicate") OR ("lithium disilicate*")) OR ("ceramics")) OR ("ceramic"))) AND ((("fracture") OR ("fracture*")) OR ("strength fracture*"))		
2.	WOS	35
Results for (((((((("occlusal veneers") OR ("occlusal veneer")) OR ("onlay")) OR ("onlays")) OR ("occlusal veneer*")) AND ("CAD/CAM")) AND ("dentin")) AND (((("lithium disilicate") OR ("lithium disilicate*")) OR ("ceramics")) OR ("ceramic"))) AND ((("fracture") OR ("fracture*")) OR ("strength fracture*")) (All Fields)		
3.	Scopus:	18
TITLE-ABS-KEY ( ( ( ( ( ( ( ( ( "occlusal veneers" ) OR ( "occlusal veneer" ) ) OR ( "onlay" ) ) OR ( "onlays" ) ) OR ( "occlusal veneer*" ) ) AND ( "CAD/CAM" ) ) AND ( "dentin" ) ) AND ( ( ( ( "lithium disilicate" ) OR ( "lithium disilicate*" ) ) OR ( "ceramics" ) ) OR ( "ceramic" ) ) ) AND ( ( ( "fracture" ) OR ( "fracture*" ) ) OR ( "strength fracture*" ) ) )		
4.	EMBASE	26
('occlusal veneers' OR 'occlusal veneer' OR 'onlay' OR 'onlays' OR 'occlusal veneer*') AND ('cad/cam'/exp OR 'cad/cam') AND ('dentin'/exp OR 'dentin') AND ('lithium disilicate'/exp OR 'lithium disilicate' OR 'lithium disilicate*' OR 'ceramics'/exp OR 'ceramics' OR 'ceramic'/exp OR 'ceramic') AND ('fracture'/exp OR 'fracture' OR 'fracture*' OR 'strength fracture*')		

### Selection criteria

The selection strategy was established according to the PICOS framework (Table 2) and followed strict inclusion criteria: in vitro experimental studies published in English, Portuguese or Spanish within the last 10 years up to 2025, evaluating the fracture resistance of lithium disilicate occlusal veneers cemented to dentin. The exclusion criteria included in vivo studies, finite element analyses, literature reviews, and case reports.

**Table 2:** PICO FORMAT

Acronim	PICO format	
P	Population	Lithium disilicate occlusal veneers CAD/CAM Keywords: “occlusal veneers”, “occlusal veneer”, “onlay”, “onlays”, “occlusal veneer*”
I	Intervention	Lithium disilicate occlusal veneers CAD/CAM bonded on dentin. Keywords: “CAD/CAM”, “lithium disilicate”, “lithium disilicate*” “ceramics”, “ceramic”, “dentin”
O	Outcome	Evidence in the literature regarding the fracture resistance of CAD/CAM-machined lithium disilicate occlusal veneers bonded to dentin. Keywords: “fracture”, “fracture*”, “strength fracture*”

### Study selection process

To obtain the final selection of articles and eliminate duplicates, the references were filtered using Rayyan software ^22^. Two reviewers (J.LL.D. and M.A.C.) independently assessed the titles and abstracts. In cases of disagreement, a third reviewer (P.L.T.L.) resolved the discrepancies and made the final inclusion decision. The inclusion and exclusion criteria were subsequently applied to select the final set of articles for analysis.

### Data recollection process

For data extraction, the following variables were considered: author, journal, year, country, testing material, study groups, veneer thickness, dental substrate, type of cement, type of aging process, type of test, measurement unit, instrument and fracture resistance values obtained.

### Risk of Bias Assessment

To assess the quality of the selected articles, the **QUIN tool (Quality Assessment Tool for In Vitro Studies)** ^23^ was used. This includes a 12-item questionnaire for verifying experimental studies (Table 3), scoring each item as: Adequately specified = 2 points, Inadequately specified = 1 point, Not specified = 0 points, Not applicable = excluded from final calculation. The final score was calculated using the formula: Final score (%) = Final score × 100 / [2 ×(x)] (x = number of applicable items). Based on this, risk of bias was categorized as: >70%: Low risk, 50-70%: Medium risk, <50%: High risk.

Two independent reviewers evaluated the articles. Any disagreements were resolved with the help of a third reviewer.

**Table 3:** QUIN Tool (quality assessment tool for in vitro studies)

# Criteria	Criteria	Details
1	Clearly stated aims/objectives	Study should clearly state aims and/or objectives, which should then be followed throughout.
2	Detailed explanation of sample size calculation	Details regarding method by which given sample size calculated should be clearly stated. Details regarding software program, formula, and parameters used for calculation of sample size should also be specified.
3	Detailed explanation of sampling technique	Details regarding predefined population from which sample has been selected. Details of sampling technique and inclusion and exclusion criteria should be clearly stated
4	Details of comparison group	Details of comparison group (positive control, negative control, or standard) should be clearly specified.
5	Detailed explanation of methodology	Clarity of procedure, method of standardization, and details of any universal standards used (if applicable) should be clearly stated.
6	Operator details	Number of operators and details regarding training and calibration of operator/s (inter-operator and intraoperator reliability) should be clearly specified.
7	Randomization	Details regarding sequence generation and allocation concealment should be clearly stated
8	Method of measurement of outcome	Clarity of procedure and rationale for choosing method should be stated. Method of standardization along with details of any universal standards used (if applicable) should also be clearly specified.
9	Outcome assessor details	Number of outcome assessors and details regarding training and calibration of assessor/s (interoutcome and intra-outcome assessor reliability) should be clearly specified.
10	Blinding	Details regarding blinding of operator(s), outcome assessor(s), and statistician should be clearly specified
11	Statistical analysis	Details regarding software program used and statistical analysis should be clearly specified.
12	Presentation of results	Outcome should be based on predefined aims and/or objectives. All data should be adequatel tabulated with baseline data clearly specified (if applicable).

## RESULTS

**Figure 1:**
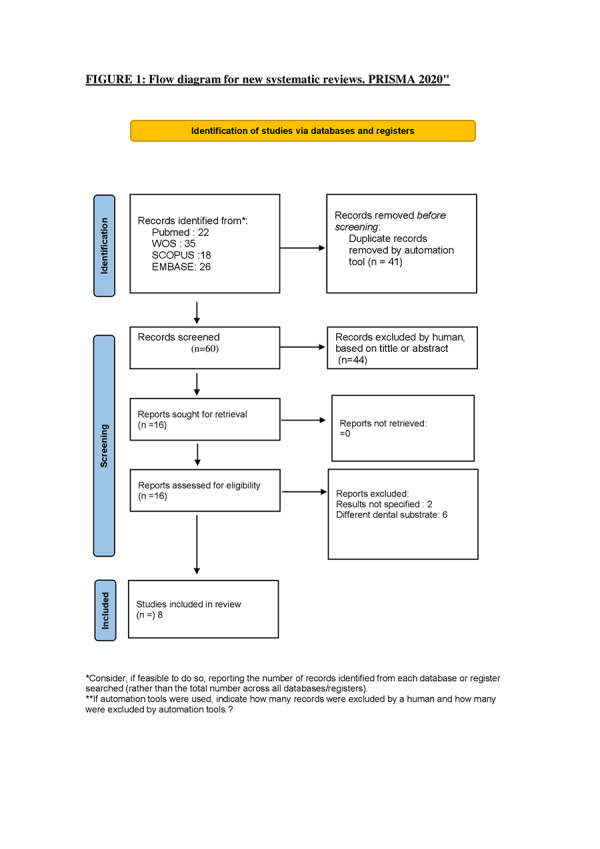
Flow diagram for new systematic reviews. PRISMA 2020"

The search strategy (Figure 1) retrieved a total of 101 articles from the selected databases. After the removal of 41 duplicates, 44 articles were excluded based on title or abstract screening, leaving 16 articles for full-text review. Of these, 8 articles were excluded for not meeting the inclusion criteria. Consequently, 8 studies were included in the final analysis (Table 4).

*Consider, if feasible to do so, reporting the number of records identified from each database or register searched (rather than the total number across all databases/registers).

**If automation tools were used, indicate how many records were excluded by a human and how many were excluded by automation tools.?

**Table 4:** Characteristics of selected articles

Autor	Journal	Year	Country	Sample Size	Prosthetic Material	Study Groups	Veneer Thickness	Dental substrate	Type of Cement	Aging Method	Type of Test	Unit of Measurement	Measuring Instrument	Measurements Obtained
Andrade et al^24^	Operative Dentistry	2017	Brazil	N=70 molars	- Lithium disilicate e.max CAD, Ivoclar Vivadent -Hybrid ceramic Vita Enamic VITA Zahnfabrik -Nanoceramic composite Lava Ultimate 3M ESPE, St Paul, MN, USA	7 groups 1) Sound theeth (control) 2) IPS e.max CAD 0.6 mm 3) IPS e.max CAD 1.5 mm 4) Vita Enamic 0.6 mm 5)Vita Enamic 1.5 mm 6) Lava Ultimate 0.6 mm 7) Lava Ultimate 1.5 mm.	0.6 mm and 1.5mm	Dentin	Variolink N (Ivoclar Vivadent,S)	Fatigue cyles: 1,000,000 on destilled water 37°	Fracture strength	Newtons	Universal testing machine DL-2000 (EMIC, Sa˜o Jose dos Pinhais, PR Brasil)	-IPS E.MAX CAD 0.6mm : 3384 N -IPS E.MAX CAD 1.5mm : 4995 N
Yazigi et al^31^	Journal of the Mechanical Behavior of Biomedical Materials	2017	Germany	N=96 premolars	- Lithium disilicate IPS e.max CAD (Ivoclar Vivadent, Schaan, Liechtenstein ) -Lithium disilicate HPR IPS e.max Press (Ivoclar Vivadent, Schaan, Liechtenstein)	3 groups of 32 1) Without inmediate dentin sealing. 2) With inmediate dentin sealing with total acid etch 3)With inmediate dentin sealing with selective acid etching. 2 subgroups (n: 16) in each group, by acid etching : total etch or selective etch.	0.8mm	Dentin	Variolink Esthetic LC; (Ivoclar Vivadent).	Storage on 37° water for 2 weeks por 2 Thermomecanic Simulador and thermocyling between 5 - 55° for 30 sec.	Fracture strength	Newtons	Willytec Kausimulator, Willytec, Munich, Germany	-Ips.e.max cad ids total etch: 1198n -Ips.e.max cad without ids with selective etch:1396 n -Ips.e.max cad ids total etch plus total etch :1777n -Ips.e.max cad with ids total etch plus selective etch: 1720 n -Ips.e.max cad ids selective with total etch :1780 n -Ips.e.max cad sdi selective plus selective etch: 1848n
Zamzam et al^25^l	Journal of the mecánicas behavior of biomedical materials	2019	USA	N=60 molars	- Hybrid ceramic Vita Enamic, - Lithium disilicate IPS e.max CAD (Ivoclar Vivadent, Liechtenstein) - Bruxzir Zirconia (Glidewell Laboratories, Irvine, USA).	3 groups of 20. 1) Vita Enamic (Vita Zahnfabrik, Badsackingen, ¨ Germany) 2) IPS e.max CAD (Ivoclar Vivadent, Liechtenstein) 3) Bruxzir (Glidewell Laboratories, Irvine, USA).	1.5mm	Dentin	Panavia F2.0, Kuraray Medical, Okayama, Japan	Storage at incubator for 3 weeks at 37°	Fracture strength	Newtons	Universal testing machine (MTS 858 Mini Bionix II, MN, USA)	IPS e. max CAD 1.5mm: 493.21 ± 102.24 N
Zhang et al^28^	Journal of Prosthodontics	2020	China	N=64 premolars	-MC: microhybdrid composite (Ceramage, Shofu Inc., Kyoto, Japan); -FMC: microhybrid fiber reinforced composite (EverStick C&B, GC, Tokyo,Japan) -Lithium disilicate :HPC: Lithium disilicate IPS e.max Press(Ivoclar Vivadent) -CCC: Lithium disilicate (IPS e.max CAD, Ivoclar Vivadent)	2 groups , divided in 4 each 1) G1: thickness 1.5 mm MC /FMC/HPC /CCC 2) G2:thickness 2.5mm MC /FMC/ HPC /CCC	1.5mm and 2.5mm	Dentin	Superbond C&B, Sun Medical, Moriyama, Japan	Thermocycling and fatigue testing: 1,200,000 cycles with 50 N and 5°C y 55°C	Fracture strength	Newtons	Universal testing machine Instron 5969,Instron, Boston, IL	CCC (IPS E.MAX CAD) : -1.5mm: 2985.64 N -2.5mm: 3066.45 N
Albelasy et al^26^	Journal of the mechanical behavior of biomedical material	2021	China	N= 84 molars	-Lithium disilicate e.max CAD -Hybrid ceramic Vita Enamic, - Nanoceramic composite Lava Ultimate	3 groupss: 1)e.max CAD : 1 and 1.5mm 2)Vita Enamic :1 and 1.5 mm 3)Lava Ultimate: 1 and 1.5mm	1 and 1 .5mm	Dentin	Rely X Unicem, 3M ESPE, St Paul, Minnesota, USA	Thermocycling of 5000 cycles at 5° y 55°	Fracture strength	Newtons	Universal testing machine Instron 3345, Canton, Massachusetts, USA	IPS e.Max CAD 1mm: 1889.96N IPS e.Max CAD 1.5mm: 2127.09N
Al-Zordk et al^27^	Materials	2021	Egypt	N= 90 molars	- Lithium disilicate -Zirconia -Polymer infiltrated ceramic	9 groups: 1)Lithium disilicate on dentin. 2)Lithium disilicate on dentin with intracoronal cavity 3)Lithium disilicate on dentin with composite 4)Zirconia on dentin 5)Zirconia dentin with intracoronal cavity. 6)Zirconia on dentin with intracoronal cavity and composite 7)Polymer infiltrated ceramic on dentin 8)Polymer infiltrated ceramic on dentin with intracoronal cavity 9)Polymer infiltrated ceramic on dentin with intracoronal cavity and composite	1 mm	Dentin	Duo-Link Universal, Bisco Inc., Schaumburg, IL, USA	Thermocycling of 5000 cycles at 5° y 55	Fracture strength	Newtons	Universal testing machine Instron Model 3345, Canton, MA, USA	-Ips e.max cad / dentin: 2251.05 n - Ips e.max cad /dentin with intracoronal cavity: 2505.91 n -Ips e.max cad /dentin with composite: 2182.53 n
Essam et al^29^	BMC Oral Health	2023	Egypt	N:42 molars	IPS e.max CAD Lithium disilicate glass ceramics IvoclarVivadent AG, Schaan, Liechtenstein	-2 groups of: thickness : 0.5 mm (n=21) or 1 mm (n=21) -3 subgroups by sufrace contitioning : (n=7), HF acid (HF-1, HF-0.5), acidulated phosphate fluoride (APF-1, APF-0.5) Monobond etch & prime (MON-1, MON-0.5)	0.5 mm and 1 mm	Dentin	Multilinik N (Ivoclar-Vivadent	Storage on water at 37° for 75 days. After a cyclic fatigue charge of 240,000 cycles.	Fracture strength	Newtons	Universal testing machine (Instron 3345, USA) (Bluehill Universal software, Instron, USA).	-Ips.e.max cad hf-0.5 mm: 1166.2 n -Ips.e.max cad hf-1 mm : 1514.6 n -Ips.e.max cad apf 0.5 mm: 962 n -Ips.e.max cad apf-1 mm: 1405.8 n -Ips.e.max cad mon0.5 mm : 1277 n -Ips.e.max cad mon1 mm: 1644 n
Paqué et al^30^	The Academy of Dental Materials	2024	Switzerland	N: 80 molars	-IPS e.max CAD (Ivoclar Vivadent, Schaan, Liechtenstein -HPR IPS e.max Press (Ivoclar Vivadent, Schaan, Liechtenstein) -Experimental lithium disilicate (Lithoz, Vienna, Austria) -PTE IPS e.max Press (Ivoclar Vivadent, Schaan, Liechtenstein)	4 groups 1)CAM: IPS e.max CAD; Ivoclar Vivadent) 2)HPR: inyected lithium disilicate with a PPMA impress mold (IPS e.max Press; Ivoclar Vivadent, Schaan, Liechtenstein); 3)3DP: 3d impress lithium disilicate (experimental lithium disilicate; Lithoz GmbH, Vienna, Austria); 4)PTE: inyected lithium disilicate disilicato with a 3d impress mold (IPS e.max Press; Ivoclar Vivadent)	0.5mm	Dentin	Variolink Esthetic LC; Ivoclar Vivadent).	Thermomecanic Simulador on water storage of 5 and 55 °	Fracture strength	Newtons	Universal testing machine (Zwick / Roell Z010; Zwick, Ulm, Germany)	-Ips.e.max cad :1746 n

All eight studies used human teeth as specimens; however, only one study employed premolars, while molars were predominantly selected for preparation and subsequent cementation of occlusal veneers ^24^^-^^31^. The selected teeth met specific parameters to minimize sample variability, including appropriate size, sound condition (unworn teeth), and standardized dimensions averaging 10.0 mm mesiodistal width and 10.5 mm buccolingual height (± 2 mm).

All studies performed dentin-level preparations, using dentin as the bonding substrate for the occlusal veneers ^24^^-^^31^.

Regarding the materials tested, all eight studies evaluated lithium disilicate veneers fabricated using CAD/CAM technology. Some studies also included comparisons with pressable lithium disilicate, nanoceramic resins, hybrid ceramics, and zirconia. The thickness of the restorations ranged from 0.5 mm to 2.5 mm, following the minimally invasive principles of occlusal veneer design ^24^^-^^31^.

Fracture resistance testing was conducted using universal testing machines, in accordance with the standards established in each respective country.

All eight studies incorporated aging procedures for the specimens. Five studies used thermocycling methods to simulate clinical conditions ^26^^-^^28^^,^^30^^,^^31^, while the remaining three employed non-thermocycling methods, such as immersion in distilled water for periods ranging from 24 hours to 3 weeks at 37 °C^(24,^^25^^,^^29^.

According to the QUIN tool assessment, two studies presented a medium risk of bias (68.18%), and six studies presented a low risk of bias (72.72%) (Table 5).

**Table 5:** Bias risk analysis according to the QUIN questionnaire tool *

AUTHORS	Clear objectives	Detailed explanation of sample size calculation	Detailed explanation of sampling technique	Details of comparison group	Detailed explanation of methodology	Operator details	Randomization	Method of Measuring outcome	Outcome assessor details	Blinding	Statistical analysis	Presentation of results	BIAS RISK ACCORDING TO THE QUIN	BIAS RISK
Andrade et al^24^	2	0	2	2	2	0	2	2	0	na	2	2	72.72%	low risk
Zamzam et al^25^	2	0	2	2	2	0	2	2	0	na	2	2	72.72%	low risk
Albelasy et al^26^	2	0	1	2	2	0	2	2	0	na	2	2	68.18%	medium risk
Al-Zordk et al^27^	2	0	2	2	2	2	0	2	0	na	2	2	72.72%	low risk
Zhang et al^28^	2	0	2	2	2	0	2	2	0	na	2	2	72.72%	low risk
Essam et al^29^	2	2	2	2	2	0	0	2	0	na	2	2	72.72%	low risk
Paqué et al^30^	2	2	2	2	2	0	0	2	0	na	2	2	72.72%	low risk
Yazigi et al^31^	2	0	1	2	2	0	2	2	0	na	2	2	68.18%	medium risk

## DISCUSSION

The decision to include only studies published within the last 10 years was based on criteria of recency, methodological validity, and technological relevance. In the field of restorative dentistry-particularly concerning materials such as lithium disilicate and CAD/CAM-fabricated occlusal veneers-substantial advances have occurred over the past decade in ceramic composition, adhesive systems, and manufacturing techniques.³²,³³ Restricting the search to this time frame ensured the comparability of results, reflected the current state of these restorative approaches, and enhanced the clinical relevance of the conclusions.

Moreover, due to the destructive nature of fracture resistance testing and the absence of standardized clinical methods for measuring fracture resistance *in vivo*, this systematic review included only *in vitro* studies. *In vitro* testing allows for the use of standardized protocols, strict control of experimental variables (such as substrate, thickness, preparation design, and luting technique), and reliable simulation of aging and fatigue processes ^17^. In contrast, available clinical studies typically report long-term survival or failure rates but do not perform destructive testing to evaluate the mechanical performance of restorative materials.

The average human bite force in the anterior region is approximately 49 N (4.9 kg), while in the posterior region it can range between 200 and 540 N (20-55 kg) ^19^. In patients with bruxism, these forces may exceed 800 N (81 kg) ^30^. Therefore, it is essential to interpret the findings of this review in light of these physiological and parafunctional loading conditions.

According to the studies by Andrade and Zhang ^24^^,^^28^ lithium disilicate CAD/CAM occlusal veneers demonstrated higher fracture resistance than other materials such as microhybrid resins and pressable lithium disilicate. Both studies reported consistent mechanical test results, showing superior fracture resistance for CAD/CAM-fabricated lithium disilicate veneers regardless of thickness, when compared to their pressable counterparts, despite identical chemical composition.

This difference may be attributed to the manufacturing process, which directly influences the material’s microstructure and mechanical behavior ^34^^-^^36^. In IPS e.max Press (pressable), the lithium disilicate crystals measure approximately 4 µm in length and 0.6 µm in width, whereas in IPS e.max CAD (CAD/CAM), the crystals are about 1 µm long and 0.4 µm wide. These differences in crystal dimensions are significant, as they directly affect the fracture resistance of ceramics ^37^.

In the study by Zamzam et al. ^25^, three CAD/CAM materials-including lithium disilicate-were evaluated using a static lateral load instead of an axial load. This testing configuration resulted in lower fracture resistance values: 493 N (50.27 kg) for lithium disilicate CAD/CAM occlusal veneers. In contrast, other studies employing axial loading reported markedly higher values, reaching up to 2356 N (240.25 kg) for veneers with a thickness of 1.5 mm ^38^.

As previously mentioned, the fracture resistance of CAD/CAM occlusal veneers is significantly influenced by both material type and thickness. In the study by Albelasy et al. ^26^, the authors also investigated the effect of different aging methods. Veneers were stored either in distilled water for 24 hours or in artificial saliva at 37 °C for six months. The study found no significant differences in average fracture resistance between storage conditions. According to the authors, this result may be attributed to the controlled industrial manufacturing of CAD/CAM blocks, which minimizes porosity and produces a more homogeneous material with reduced water sorption ^37^, thereby improving mechanical performance.

While many studies emphasize the restorative material, it is equally important to consider the type of cement used. For instance, in the study by Al-Zordk et al. ^27^, mechanical testing was conducted using different CAD/CAM materials, including lithium disilicate. The results showed no significant differences in fracture resistance between lithium disilicate, zirconia, and resin-infiltrated ceramic veneers; however, the resin-infiltrated ceramic group exhibited the highest values. This behavior may be explained by the similar elastic moduli of resin-infiltrated ceramics and dentin.

Nevertheless, other studies have reported lower fracture resistance values for this material, possibly due to variations in adhesive protocols, such as the use of self-etch primers. The fracture resistance values for lithium disilicate veneers in that study were consistent with other findings reported in the literature ^38^^,^^39^.

In addition to material and thickness, the bonding substrate is another critical variable. The present review included only studies in which occlusal veneers were bonded to dentin, as this condition represents a common clinical scenario in patients presenting with moderate tooth wear.

In the study by Essam et al. ^29^ the surface conditioning method prior to cementation was also evaluated. Hydrofluoric acid (HF) is known to enhance bonding to lithium disilicate; however, previous studies have reported that, depending on its concentration and exposure time, HF may negatively affect the mechanical strength of ceramics ^40^^-^^41^. The authors tested three conditioning agents: acidulated phosphate fluoride (APF), which acts more superficially and is commonly used in orthodontics; hydrofluoric acid (HF); and Monobond Etch & Prime (MON). Regardless of the conditioning agent, increased restoration thickness (1 mm vs. 0.5 mm) yielded higher fracture resistance. Even 0.5 mm restorations exceeded average molar bite forces (400-800 N) ^29^.

It is crucial to follow a strict conditioning protocol for lithium disilicate to ensure optimal bonding. But it's also important to enhance the condition of the bonding substrate. For this reason, Yazigi et al. ^31^ investigated the effect of immediate dentin sealing (IDS). Specimens treated with IDS exhibited significantly higher fracture resistance than those without. According to the authors, this improvement is likely due to the pre-curing of the adhesive, which creates a more uniform hybrid layer with resin tags sealing the dentinal tubules. This configuration reduces stress on the dentin during cementation and allows occlusal forces to be distributed more evenly-functioning as a monoblock-which explains the observed increase in fracture resistance.

In the study by Paqué et al. ^30^ the authors compared veneers fabricated using subtractive (CAD/CAM) and additive (3D printing) techniques. Although no significant differences in fracture resistance were observed, notable differences were found in marginal fit between pressed and CAD/CAM lithium disilicate restorations ^24^.

Based on the evidence reviewed, the ideal thickness for occlusal veneers appears to range between 0.8 mm and 1.0 mm for optimal performance. While thinner restorations (<0.6 mm) may present processing-related failures, they still demonstrate high fracture resistance.

Further research is recommended to explore additional variables related to the adhesive phase, with the goal of optimizing cementation protocols-particularly for bonding to dentin, which exhibits lower adhesion potential than enamel. Refining these protocols will contribute to improved clinical performance and long-term success of occlusal veneers.

## CONCLUSIONS

• CAD/CAM lithium disilicate occlusal veneers bonded to dentin exhibit optimal fracture resistance values, exceeding those expected under high occlusal load conditions in clinical settings.
• The recommended thickness for lithium disilicate CAD/CAM occlusal veneers is between 0.8 mm and 1.0 mm, with studies showing that a thickness of 1.5 mm can double the fracture resistance values.
• Immediate dentin sealing (IDS) prior to cementation has been associated with higher fracture resistance, suggesting that this technique enhances the mechanical behavior and durability of restorations bonded to dentin.
